# Evaluation of the Effect of the Inspired Oxygen Fraction on Blood Oxygenation during Inhalant Anaesthesia in Horses: A Systematic Review with Meta-Analysis

**DOI:** 10.3390/ani11082245

**Published:** 2021-07-30

**Authors:** Ioannis Savvas, Kiriaki Pavlidou, Christina Braun, Stijn Schauvliege, Francesco Staffieri, Yves Moens

**Affiliations:** 1 Companion Animal Clinic, School of Veterinary Medicine, Aristotle University of Thessaloniki, 546 27 Thessaloniki, Greece; kellypav@gmail.com; 2 Anaesthesiology and Perioperative Intensive Care Medicine, University of Veterinary Medicine, 1210 Vienna, Austria; Christina.Braun@vetmeduni.ac.at (C.B.); yves.moens@vetmeduni.ac.at (Y.M.); 3 Department of Large Animal Surgery, Anaesthesia and Orthopaedics, Faculty of Veterinary Medicine, Ghent University, 9820 Merelbeke, Belgium; Stijn.Schauvliege@UGent.be; 4 Section of Veterinary Clinics and Animal Production, Department of Emergency and Organs Transplantation, University of Bari Aldo Moro, 70010 Bari, Italy; francesco.staffieri@uniba.it

**Keywords:** anaesthesia, blood oxygenation, horses

## Abstract

**Simple Summary:**

In anaesthetized horses, blood oxygenation impairment often occurs. This systematic review compared the effects of low and high inspired oxygen fractions on the arterial oxygen tension and other pulmonary gas exchange parameters in horses during inhalation anaesthesia. Five studies, four experimental and one clinical, were deemed suitable for inclusion. A meta-analysis was performed on the four experimental studies. The oxygen partial pressure was significantly lower with a lower inspired oxygen fraction. However, indices of pulmonary gas exchange were significantly worsened. It is concluded that, while only a limited number of studies are available, the use of a higher inspired oxygen fraction in horses during inhalation anaesthesia will result in higher levels of oxygen in the blood; it will also worsen the lung gas exchange status. Further studies are needed to increase the level of evidence on this subject.

**Abstract:**

In anaesthetized horses, pronounced ventilation/perfusion mismatching often occurs. Several authors have investigated the effect of lower inspired oxygen fractions (FiO_2_) to reduce formation of absorption atelectasis. This systematic review compared the effects of low (<0.6) and high (>0.8) FiO_2_ on the arterial oxygen tension (PaO_2_), the alveolar-to-arterial oxygen tension difference (P(A-a)O_2_), and the PaO_2_/FiO_2_ ratio in horses during inhalation anaesthesia. Using the Systematic Review Protocol for Animal Intervention Studies, four experimental and one clinical investigations were deemed suitable for inclusion. A meta-analysis was performed on the four experimental studies. The PaO_2_ was significantly lower (*p* = 0.0007, mean difference −23.54 kPa, 95% CI −37.18, −9.90) with a lower FiO_2_. However, the P(A-a)O_2_ was also significantly lower (*p* < 0.00001, mean difference −20.80 kPa, 95% CI −26.28, −15.32) when using a low FiO_2_. For the PaO_2_/FiO_2_ ratio, only one study fitted the inclusion criteria, so no meta-analysis was performed. It is concluded that, while only a limited number of studies are available, the use of a higher FiO_2_ in horses during inhalation anaesthesia will result in higher levels of PaO_2_, but also a larger P(A-a)O_2_ difference. Further studies are needed to increase the level of evidence on this subject.

## 1. Introduction

Since almost sixty years ago, studies in humans have shown that during halothane anaesthesia with spontaneous breathing, ventilation/perfusion relationship (V/Q) abnormalities and intrapulmonary shunt may develop, resulting in a reduction in the arterial partial pressure of oxygen (PaO_2_). One of the main causes of impaired oxygenation of the blood seems to be the development of atelectasis during anaesthesia, which reduces lung compliance and PaO_2_. There is supporting evidence that this condition may develop in humans, horses, and other animal species [1]. This was confirmed in computed tomography (CT) studies in humans, which revealed atelectasis of the most dependent parts of the lungs in 90% of the anaesthetised patients [2,3]. Development of atelectasis is considered to happen immediately after induction of anaesthesia [4,5]. Lung compression, gas absorption, and surfactant impairment are the major causative factors for atelectasis development [2].

Because of these reasons, it has been assumed that an alveolar partial pressure of oxygen (PaO_2_) of at least 26.6 kPa (200 mmHg) is needed in order to preserve normal PaO_2_ values [6,7,8]. However, higher levels of the fraction of inspired oxygen (FiO_2_) will increase the rate of gas absorption from partially or completely occluded alveoli and produce atelectasis [2,6]. In fact, the use of FiO_2_ 1.0 may even be the major causative factor for atelectasis development [4,9,10,11,12], since the composition of the inspired gas is directly related to the rate of the alveolar collapse of a completely closed lung unit.

In animals, lower PaO_2_ values have also been recorded in patients intraoperatively than in conscious subjects breathing the same FiO_2_. As in humans, a major factor for this is V/Q alterations [1]. In horses, pronounced V/Q abnormalities are commonly found, mainly caused by atelectasis formation in the dependent lung regions [13], a condition first published in an original and seminal paper on the subject, wherein it was shown that under halothane anaesthesia, a severe reduction in pulmonary ventilation may develop in horses [14]. However, the use of FiO_2_ 1.0 to compensate for the atelectatic areas [15] may itself result in severe pulmonary atelectasis, creating a controversy among clinicians regarding the optimal FiO_2_.

Evidence from studies in animals (dog [16], cat [17], sheep [18], and horse [19,20,21]) indicate that the use of a low FiO_2_ may be beneficial in reducing lung atelectasis. On the other hand, there is evidence that FiO_2_ 0.3 [22] or 0.5 [23] does not improve arterial oxygenation or gas exchange compared to FiO_2_ above 0.9. Some studies evaluate aeration based on the CT images of the lungs for atelectasis formation, while others investigate the oxygenation status of the animals.

Since there is lack of supporting evidence for the best FiO_2_ values intraoperatively in horses, we conducted this systematic review. The objective of our review was to systematically identify, appraise, and synthesise the evidence in relation to different FiO_2_ levels (high or low) in horses anaesthetised with inhalant anaesthetics. Specifically, our PICO question was: “Does a reduced FiO_2_ (below 0.6) compared to FiO_2_ above 0.8 improve blood oxygenation in horses during anaesthesia?”.

## 2. Methods

A study protocol was established using the Systematic Review Protocol for Animal Intervention Studies (SYRCLE) [24].

### 2.1. Type of Studies

We included controlled studies on either experimental or client-owned animals, which compared at least two different FiO_2_ values during inhalant anaesthesia in horses. Reviews were excluded. Only publications in the English language were evaluated.

### 2.2. Population/Species Studied

The target species was the horse, of all ages. Only normocapnic patients were included.

### 2.3. Interventions

For the purpose of this review, a standard (control) treatment was defined as an FiO_2_ more than 0.8 and the intervention/exposure treatment as an FiO_2_ below 0.6. The mixture of inspired gas should contain medical air or nitrogen, but not other gases.

### 2.4. Outcome Measures

- Arterial partial pressure of oxygen (PaO_2_). All values were transformed to kPa.
- Alveolar–arterial difference in the partial pressure of oxygen (P(a-a)O_2_). All values were transformed to kPa.
- Arterial partial pressure of the oxygen to fraction of inspired oxygen ratio (PaO_2_/FiO_2_).

### 2.5. Search Method

Four electronic databases were searched:

- MEDLINE via PubMed;
- Web of Science/CAB Abstracts;
- SCOPUS.

The search string was:

(“oxygen”) AND (“oxygenation” OR “atelectasis” OR “gas exchange” OR “oxygen tension” OR “pressure of oxygen” OR “oxygen partial”) AND (equine* OR horse*) AND (anaest* OR anest*)


This string was adapted according to the search rules/code of the database used. All dates of publication were searched until end of May 2021.

### 2.6. Selection of Studies

Two groups, with two persons each (I.S. and K.P., and C.B. and S.S.), screened the results of the search output. Discrepancies were resolved with collaboration and critical discussion between the two groups. The first selection phase consisted of the evaluation of the title and abstract of the studies. The studies selected in this phase passed onto the second phase—the critical reading of the full paper. Whenever the authors of this review were also authors of an eligible study or had been a reviewer thereof, they were excluded from the evaluation.

### 2.7. Data Extraction and Management

Details of the eligible studies were independently extracted by the two groups of reviewers. Data extracted were:

- Authors, title, year of publication, and journal;
- Number of animals in intervention and control groups;
- Horses, age, weight, status ASA, inhalant agent, and spontaneous/mechanical ventilation;
- Outcome measures;
- Presence of any other outcome measures;
- Excluded animals (dropouts).

### 2.8. Assessment of Risk of Bias in the Included Studies

The two groups of reviewers assessed the included studies using the SYRCLE’s Risk of Bias tool [25]. The following details were agreed on: When the study was randomised, but there was no mention of the randomisation method, we judged the study to have an unclear risk of bias. Random housing of the experimental animals was judged as low or unclear, as well as the animal assessors and animal selection blindness, because we assumed that these were mostly irrelevant to our review.

### 2.9. Data Analysis

Data were introduced into a specific software (Review Manager/RevMan Version 5.3. Copenhagen: The Nordic Cochrane Centre, The Cochrane Collaboration, 2014), where they were stored, analysed, and synthesised for the production of the meta-analysis. All three outcomes were continuous variables and were analysed with the inverse variance method with a random effects model. No subgroup analysis was performed (e.g., inhalant agent, and recumbency), because of the small number of the included studies. Effect measures are presented as the mean differences. Heterogeneity and overall effects were calculated. Statistical significance was set to α = 0.05.

## 3. Results

**Figure 1:**
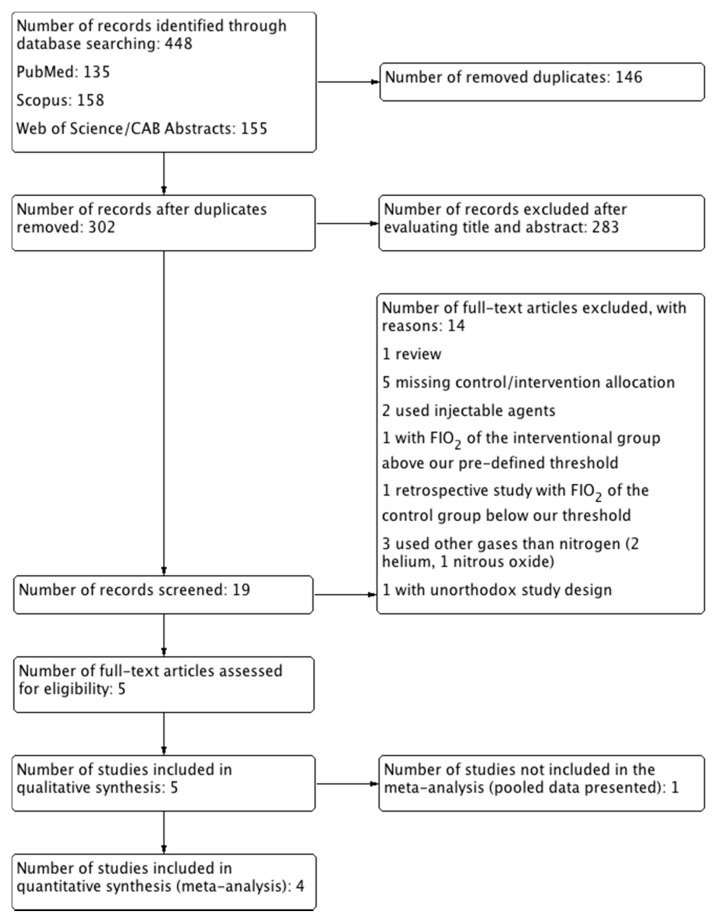
Study flow diagram.

A total of 448 papers were retrieved. A PubMed search returned 135 results, Scopus 158, and Web of Science/CAB Abstracts 155. After removing the duplicates, 302 papers remained. The first selection phase revealed 19 papers eligible for further evaluation. The second selection phase revealed 5 papers, which were included in this review, and 14 papers were excluded (Figure 1).

### 3.1. Characteristics of the Included Studies

Four studies were experimental, and one study was a clinical trial. In the experimental studies, isoflurane was used to maintain anaesthesia in three studies (two with mechanical ventilation [23,26] and one with spontaneous ventilation [27]), and halothane in one (spontaneous ventilation [19]). In the clinical trial, isoflurane was used for the maintenance of anaesthesia with mechanical ventilation [28] (Table 1).

All the experimental studies had a crossover design, with 5–8 horses included in each one. Three were adequately randomised, but one had an unclear risk of randomisation bias [19]. The clinical study was a prospective randomised one. Outcome measures were serially (over time of anaesthesia) recorded in all studies. We collected and analysed data for the meta-analysis at the 90 min timepoint from all the experimental studies, which was the commonest timepoint among the studies. In the clinical study [28], data had been recorded at three timepoints during anaesthesia, and the pooled data are presented in the paper; therefore, the data were extracted for the qualitative analysis, but were not used for the meta-analysis.

In all studies, the standard treatment was an FiO_2_ above 0.85. Intervention treatment was an FiO_2_ of 0.5 in two experimental studies. In the other three studies, the intervention groups received an FiO_2_ of 0.21 (experimental study) and 0.3 (one experimental and one clinical).

In only one study [28] an a priori power analysis was performed, however the post hoc power of the study was poor.

### 3.2. Risk of Bias of the Included Studies

**Figure 2:**
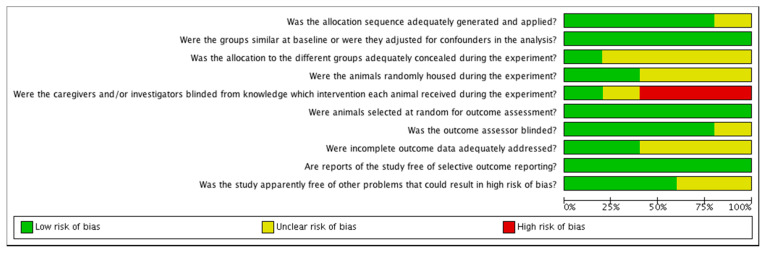
Risk of bias graph: review authors’ judgements about each risk of bias item presented as percentages across all included studies.

**Figure 3:**
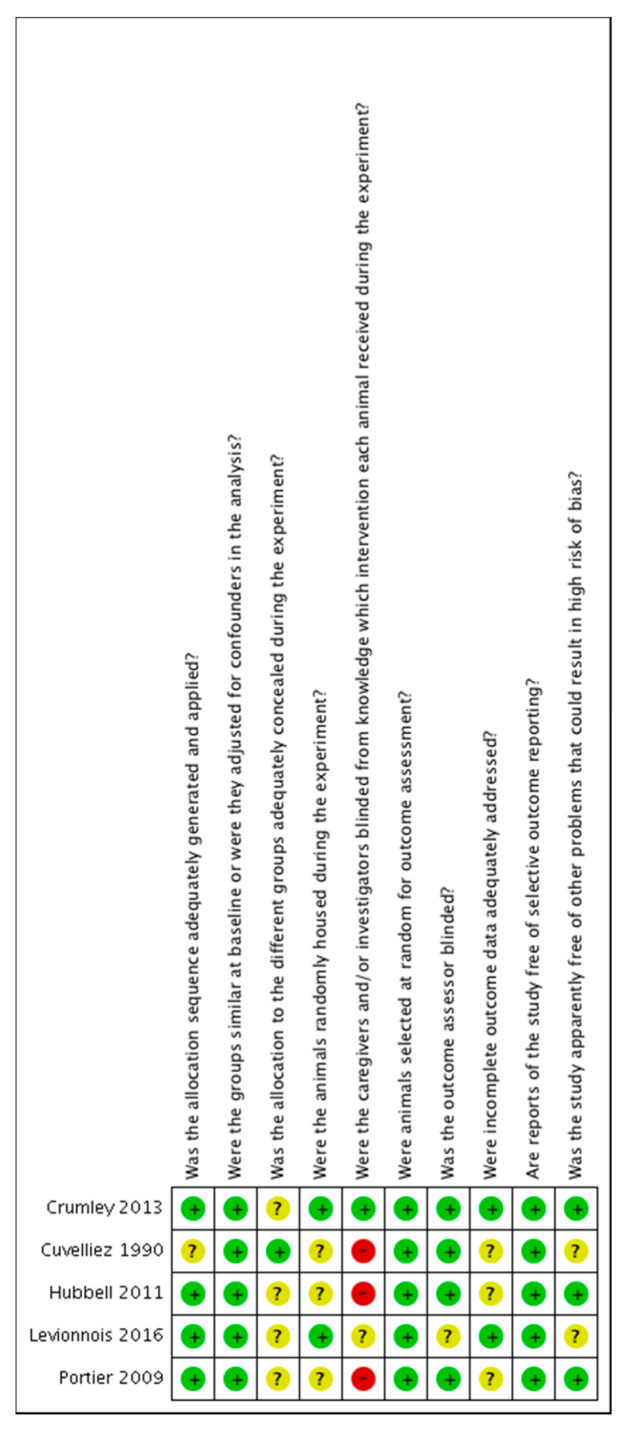
Risk of bias summary: review authors’ judgements about each risk of bias item for each included study. ?: unclear risk; −: high risk; +: low risk.

The risk of bias was found to be unclear or low in most of the studies. The risk of bias tables are shown in Figure 2 and Figure 3.

### 3.3. Characteristics of the Excluded Studies

Fourteen studies were excluded after critically evaluating them: one review [29]; five due to missing control/intervention allocation of the animals [30,31,32,33,34]; two studies [20,35] because injectable agents were used for the maintenance of anaesthesia; one with an FIO_2_ of the interventional group above our pre-defined threshold of 0.6 [36]; and one retrospective study with an average FIO_2_ of the control group below our threshold of 0.8 [37]. Furthermore, three more studies were excluded as other gases than nitrogen were used to decrease the FIO_2_: in two studies [21,38] the intervention group received a mixture of oxygen with helium, and in one study [39] the inspired mixture consisted of oxygen and nitrous oxide. Finally, one study [40] had an unorthodox study design: 24 animals were used to compare the influence of a delivered oxygen fraction (FdO_2_) of 1.0 and 0.6 during isoflurane anaesthesia. Sixteen horses underwent an arthroscopy in dorsal recumbency and were equally and randomly allocated over the two treatments, while the remaining eight horses received both treatments in a randomized crossover study in lateral recumbency for a wound healing study. The main reason for exclusion of this study is that the authors targeted a fixed FdO_2_, which may have resulted in some variability of the FiO_2_ among individual horses, although a similar influence would be expected with both treatments.

### 3.4. The Effect of Low FiO_2_ on PaO_2_

**Figure 4:**
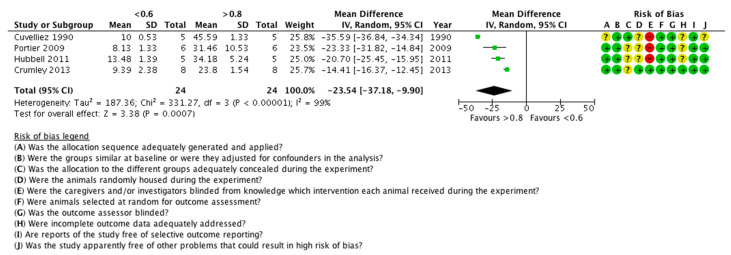
Forest plot of the effect of the FiO_2_ on PaO_2_. Units are in kPa. CI: confidence interval; IV: inverse variance; SD: standard deviation. ?: unclear risk; −: high risk; +: low risk.

Data for the FiO_2_ were extracted from all five studies; in all of them, a low FiO_2_ statistically significantly reduced the PaO_2_ of the horses. Four studies were included in the meta-analysis (Figure 4). Data from a total of 24 animals were analysed. The heterogeneity of the studies was statistically significant (*p* < 0.00001, I^2^ = 99%) and the overall effect was statistically significant (*p* = 0.0007, mean difference = −23.54, 95% CI −37.18, −9.90), in favour of the high FiO_2_.

Because of the high heterogeneity, a sensitivity analysis was also performed, by removing each study from the model. By removing the studies of Portier et al. (2009), Hubbell et al. (2011), or Crumley et al. (2013), there was a minor change in the I^2^ and the *p*-values. However, when removing the Cuvelliez et al. (1990) study, the I^2^ was reduced to 78% (still significant, *p* = 0.01), with an overall effect again statistically significant (*p* < 0.00001, mean difference = −18.58, 95% CI −24.26, −12.91), in favour of the high FiO_2_ (19 animals in the model). It seems that the Cuvelliez et al. (1990) study is the major source of heterogeneity; as can be seen, it has the largest mean difference between the two groups.

### 3.5. The Effect of Low FiO_2_ on P(a-a)O_2_

**Figure 5:**
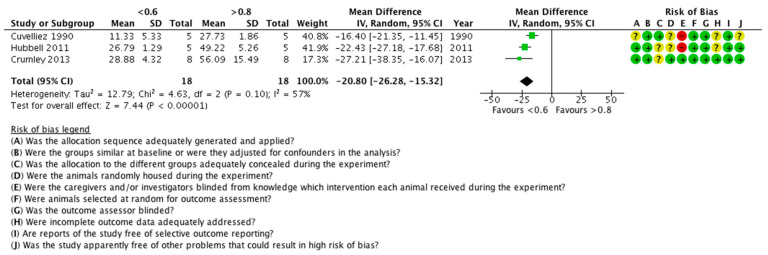
Forest plot of the effect of FiO_2_ on P(a-a)O_2_. Units are in kPa. CI: confidence interval; IV: inverse variance; SD: standard deviation. ?: unclear risk; −: high risk; +: low risk.

Data of P(a-a)O_2_ measurements were found in four studies. In one study [28], there was a statistically non-significant difference between the control and the intervention groups, regarding the P(a-a)O_2_, but these data were not included in the meta-analysis, because they were pooled out of several timepoints. In the remaining three studies, the low FiO_2_ statistically significantly reduced the P(a-a)O_2_ (Figure 5). Data from a total of 18 animals were analysed. The heterogeneity of the studies was statistically non-significant (*p* < 0.1, I^2^ = 57%) and the overall effect was statistically significant (*p* < 0.00001, mean difference = −20.80, 95% CI (−26.28, −15.32)), in favour of the low FiO_2_.

### 3.6. The Effect of Low FiO_2_ on PaO_2_/FiO_2_

Data on PaO_2_/FiO_2_ measurements were found in two studies. In one study [28], there was a non-significant difference in PaO_2_/FiO_2_; however, these data were not used for the meta-analysis, because they were pooled out of several timepoints. In the other study [23], the low FIO_2_ statistically significantly reduced the PaO_2_/FIO_2_. No meta-analysis was produced for this outcome.

## 4. Discussion

According to our knowledge, this is the first systematic review with a meta-analysis to evaluate the effect of the inspired oxygen fraction on the oxygenation of the blood in anaesthetised horses. This review combined the results of five studies, four experimental and one clinical (not used in the meta-analysis), including a total of 64 horses (24 in the meta-analysis). It is interesting that very few clinical studies were found, although we believe that the extent of the use of low FiO_2_ mixtures in clinical practice is higher than depicted in the published literature; an indication is given in a retrospective study [37]. In an attempt to have more meaningful results, we excluded studies with injectable agents, because the effect of the different injectable anaesthetic drugs on the respiratory system and the pulmonary function is diverse.

Moreover, we excluded studies where the inspired gas mixture consisted of gas other than oxygen with medical air or nitrogen. For instance, we excluded two studies with helium in the inspired mixture [21,38], because of the specific physical properties of helium. Interestingly, in both studies, PaO_2_ and P(a-a)O_2_ were higher (P(a-a)O_2_ (non-significantly in [38]) with a high FiO_2_, whereas the PaO_2_/FiO_2_ ratio was lower. Moreover, we excluded another study [39] with N_2_O in the mixture, an agent with special anaesthetic and analgesic properties, which may have affected pulmonary function. In this study, PaO_2_ was also higher in high FiO_2_. Although we have not included these three studies in our review, we assume that this has not substantially affected our results.

A variety of factors, e.g., breed, gender, age, type of ventilation, application of positive end-expiratory pressure, tidal volume used, etc. [1], as well as body position, body weight, and thoracic conformation [41,42] may affect the oxygenation of the blood. Because of the diversity of the equine population recruited in these studies, we assumed that the heterogeneity of the studies was high, and it was treated as such in the meta-analysis (random effect analysis was used). That was also the reason why a subgroup analysis was not performed.

As an appraisal method to estimate bias, we used the SYRCLE’s risk of bias tool for animal studies, which is an adapted version of the Cochrane risk of bias tool. Although it is not fully validated, it takes into account the specific aspects of the experimental design of animal studies compared to clinical studies [25]. In our review, we have included experimental as well as randomised clinical animal studies, so we believe that the choice of the SYRCLE’s risk of bias tool was the best available option.

From this review, it is clear that the arterial partial pressure of the oxygen is higher when a high oxygen fraction is inhaled, which is an expected result, especially when mechanical ventilation is applied. However, this is not the only index of oxygen exchange. The horse may not be hypoxic, although severe intrapulmonary V/Q mismatch may develop intraoperatively, and lead to increased mortality post-operatively [15]. Furthermore, in practice, as long as the PaO_2_ is in the range to fully saturate haemoglobin, differences in PaO_2_ have limited relevance. It may, e.g., be more useful to study the influence of the FiO_2_ on the incidence of hypoxaemia. Other indices are also used to assess pulmonary gas exchange. P(a-a)O_2_ is an index of intrapulmonary shunt or V/Q scatter, although it can be affected by PaO_2_, cardiac output, body temperature, pH and base excess of the blood, haemoglobin concentration, and alveolar ventilation [7]. This review revealed that the alveolar–arterial difference is higher in high fractions of inspired oxygen, an indication of compromised pulmonary function. Unfortunately, there is very limited information of the effect of high inspired oxygen on another index, the PaO_2_/FiO_2_ ratio. It would be interesting to have data of this outcome measure, since it has been shown in humans that the PaO_2_/FiO_2_ ratio, as well as the P(a-a)O_2_ difference, depends on FiO_2_ [43], whereas in sheep, the P(a-a)O_2_ difference correlation to shunt seems to be weaker than that of the PaO_2_/FiO_2_ ratio [44].

Another technique to detect the matching between alveolar ventilation and pulmonary blood perfusion is the multiple inert gas elimination technique (MIGET), which uses six (usually) inert gases [45,46]. One study [20] using this technique in horses was found during our literature search. This study shows an increased intrapulmonary shunt when high FiO_2_ is administered, despite the high PaO_2_ measured. This shunt persisted into recovery. These results support the findings of this review, although unfortunately we could not include this study in the systematic review and meta-analysis, because this experimental study used dissociative anaesthesia, and also did not calculate the indices we were looking for.

It is known that the alveolar O_2_ concentration affects the development of absorption atelectasis; however, in horses, compression of the lungs because of the shape and the position of the diaphragmatic dome, in combination with a high pressure exerted by the abdominal contents (especially in dorsal recumbency), may promote compression atelectasis. Thus, lowering the FiO_2_ may not be the only intervention to improve oxygenation, and other strategies, e.g., alveolar recruiting strategies, may be more effective in improving oxygenation.

Our review possesses some limitations. The first one is the small number of included studies and animal population. It seems that very few studies have been performed on that topic, and the ones available often differ substantially in their methodology and described outcome, making it questionable to combine their results. Thus, this systematic review is accompanied by a meta-analysis with a fairly limited number of studies. While limited data as well as high heterogeneity are reasons to avoid a meta-analysis, there is no agreement on a cut off regarding these factors [47]. In our opinion, the meta-analysis serves as an additional aid to evaluate the presented information in a concise fashion. Certainly, there is a need for more research in this area, because equine intraoperative hypoxia is a serious problem in clinical practice. The second limitation is that the subgroup analysis was not possible for any comparison. Future well-designed experimental studies and clinical trials using targeted evaluating tools are necessary to increase the level of evidence, for better decision making. The third limitation (although not an inherent one of our review, rather than a limitation of the included studies) is the lack of power analysis in the included studies, which may have compromised the level of evidence. Having in mind these limitations, and despite a clear trend towards specific results, the findings of this review should be interpreted cautiously.

## 5. Conclusions

Considering a low to medium level of evidence, the reduction of FiO_2_ in horses under anaesthesia may improve some oxygenation indices, e.g., shunt, but will decrease blood oxygenation.

## Figures and Tables

**Table 1:** Characteristics of the five (5) included studies, by first author alphabetical order and year of publication.

Reference	Type of Study/Design	Animals (Horses)	Control/Intervention	Outcomes	Recumbency	Maintenance of Anaesthesia/Type of Ventilation	Notes
Crumley 2013 [27]	experimental/randomised crossover	8 (6 geldings, 2 mares), median age 10 years), median weight 526 kg	FiO_2_ > 0.95/0.5	haemodynamic variables, blood gas analysis, oxygen indices, respiratory parameters	dorsal	isoflurane (ET 1.5%)/spontaneous	serial time measurements, data extracted at 90 min timepoint
Cuvelliez 1990 [19]	experimental/crossover	5 (2 mares, 3 geldings), various ages, mean weight 455 ± 52 kg	FiO_2_ > 0.85/0.3	haemodynamic variables, blood gas analysis, oxygen variables calculation	left lateral	halothane (ET 1.2%)/spontaneous	serial time measurements, data extracted at 90 min timepoint
Hubbell 2011 [23]	experimental/randomised crossover	5 (2 geldings, 3 mares), mature, mean weight 614 kg	FiO_2_ > 0.95/0.5	haemodynamic variables, blood gas analysis, oxygen variables calculation	dorsal	isoflurane (ET 2%)/mechanical	serial time measurements, data extracted at 90 min timepoint
Levionnois 2016 [28]	clinical/prospective randomised	40 warmbloods, various ages and weights	FiO_2_ > 0.95/0.3	haemodynamic variables, blood gases, oxygen indices, ventilatory variables	19 lateral, 21 dorsal	isoflurane (ET 1.2%)/mechanical	data collected at 3 timepoints in first hour, pooled data are presented, no data extracted for meta-analysis
Portier 2009 [26]	experimental/randomised crossover	6 (geldings), age 4.5–9.5 years, weight 510–640 kg	FiO_2_ 1.0/0.21	haemodynamic variables, blood gas analysis, oxygen variables calculation	left lateral	isoflurane, mechanical	serial time measurements, data extracted at 90 min timepoint

ET: end-tidal.

## Data Availability

Data is contained within the article.
